# Characterizations of Gene Alterations in Melanoma Patients from Chinese Population

**DOI:** 10.1155/2020/6096814

**Published:** 2020-01-30

**Authors:** Yi Luo, Zhenzhen Zhang, Jianfan Liu, Linqing Li, Xuezheng Xu, Xinyu Yao, Zixun Dai, Xin Wang, Shuo Yang, Hongwei Wu, Jie Bu, Yuan Wu, Tianmin Xiang, Xianan Li

**Affiliations:** ^1^Department of Orthopedics & Soft Tissue, Hunan Cancer Hospital and the Affiliated Cancer Hospital of Xiangya School of Medicine, Central South University, Changsha, Hunan 410013, China; ^2^Singlera Genomics Inc., Shanghai 201318, China

## Abstract

Melanoma is a human skin malignant tumor with high invasion and poor prognosis. The limited understanding of genomic alterations in melanomas in China impedes the diagnosis and therapeutic strategy selection. We conducted comprehensive genomic profiling of melanomas from 39 primary and metastatic formalin-fixed paraffin-embedded (FFPE) samples from 27 patients in China based on an NGS panel of 223 genes. No significant difference in gene alterations was found between primary and metastasis melanomas. The status of germline mutation, CNV, and somatic mutation in our cohort was quite different from that reported in Western populations. We further delineated the mutation patterns of 4 molecular subgroups (BRAF, RAS, NF1, and Triple-WT) of melanoma in our cohort. BRAF mutations were more frequently identified in melanomas without chromic sun-induced damage (non-CSD), while RAS mutations were more likely observed in acral melanomas. NF1 and Triple-WT subgroups were unbiased between melanomas arising in non-CSD and acral skin. BRAF, RAS, and NF1 mutations were significantly associated with lymph node metastasis or presence of ulceration, implying that these cancer driver genes were independent prognostic factors. In summary, our results suggest that mutational profiles of malignant melanomas in China are significantly different from Western countries, and both gene mutation and amplification play an important role in the development and progression of melanomas.

## 1. Introduction

Melanoma is a type of malignant tumor that typically occurs in the human skin with high invasion, high metastasis, and poor prognosis [1]. For melanomas at early stages, surgical resection of primary melanoma can give curative effects for patients. But in cases of distant metastatic melanomas, a really poor prognosis is shown and the 5-year survival rate is less than 5% [2]. Furthermore, it is largely refractory to the standard conventional chemotherapy with a low response rate and considerable toxicities [1]. Therefore, understanding the molecular pathogenesis of melanoma is of great significance for early diagnosis and appropriate treatment of these melanoma patients.

About 70% of melanoma patients in the Asian population are diagnosed as acral and mucosal type [3], compared with 5% in European and American populations [4], of which cutaneous melanoma is the major subtype. The difference in subtypes might indicate a discrepancy of major genetic variation patterns between Asian and Caucasian melanoma patients and could direct to different therapeutic treatments. For example, while BRAF gene mutation is one of the most frequently mutated genes in European and American populations (above 40%) [5, 6], only about 25% of Asian patients harbored BRAF alterations [7]. The disparities in subtypes also have been associated with several melanoma predisposition genes. Approximately 10% of cutaneous melanoma cases occur with a strong family history [8]. About 45% of melanomas have been attributed to germline mutation of predisposition gene CDKN2A [9], and 2-3% melanomas mutated in gene CDK4 in European and American populations [10]. However, acral melanoma has not been related to the melanoma predisposition gene [11]. Oncogenomic studies indicate that somatic CNV of some genes is involved in melanoma progressions, such as mutated gene amplifications in CCND1, CDK4, and TERT [6, 12–14], of which the CNV status also has been reported in Chinese melanomas [14, 15].

Accurate classification of the spectra of mutational changes in melanoma may facilitate the development of disease-associated biomarkers. The Cancer Genome Atlas [16], based on the NGS data of 318 cutaneous melanoma samples, classified melanomas into 4 distinct subgroups: BRAF^mut^, RAS^mut^, NF1^mut^, and Triple-WT (lack of mutations in all three genes), which is currently being used in clinical practice. Studies have revealed that the RAS/RAF/MEK/ERK signaling pathway plays a critical role in melanoma development, of which ERK is activated in up to 90% of melanomas [17]. In melanoma, ERK activation is most commonly due to the mutations of BRAF, followed by RAS, NF1, and other genes [18]. In the Triple-WT subtype, alterations in genes such as KIT, FDGFRA, KDR, GNAQ, and GNA11 play an important role in melanoma carcinogenesis [2, 16, 18, 19]. The mutated or amplified genes of KIT, FDGFRA, and KDR, encoding a receptor tyrosine kinase (RTK) of the cell membrane, may activate the downstream signaling pathways in both ERK and PI3K.

The vast majority of reports about melanoma mainly come from Western countries. Relatively less of the mutation spectra are known for melanoma in Asian, and especially in China. Therefore, we conducted a genetic analysis of malignant melanomas from patients in China to determine the genomic landscape of this tumor.

## 2. Materials and Methods

### 2.1. Patients and Samples

A total of 39 formalin-fixed, paraffin-embedded (FFPE) samples including 22 primary tumors and 17 metastatic tumors were collected from 27 malignant melanoma patients in Hunan Cancer Hospital during 2016–2018. At the first diagnosis, 15 of our cohort were acral melanomas situated on palms (1 patient) and soles (14 patients), 11 were cutaneous melanomas without chronic sun-induced damage (non-CSD), and 1 patient missed pathological information. Clinicopathological information was presented in Table 1. Peripheral blood or normal tissue sample was collected from 27 cases as control. The present study was approved by the Ethics Committee of Hunan Cancer Hospital. All patients enrolled in this study provided written consent.

### 2.2. DNA Extraction from FFPE Samples

DNA from FFPE samples was extracted using the QIAamp DNA FFPE Tissue Kit (Qiagen, Germany) according to the manufacturer's instructions. DNA from blood was extracted using the QIAamp DNA Blood Maxi Kit (Qiagen). Qubit Fluorometer (Thermo Scientific, USA) was used to quantify the DNA extracted. DNA samples with more than 50 ng where the most fragments were located above 500 bp could be used in subsequent processes.

### 2.3. Library Construction and Sequencing

NGS library generating was performed under the OncoAim™ cancer Kit (Singlera Genomics, Inc., Shanghai, China), of which the gene panel included covers 1300 exons, 465 hotspot sites, 21 introns, and one promoter of 223 genes (Supplementary Table ([Supplementary-material supplementary-material-1])). SNV (single nucleotide various), InDel, CNV, and gene fusion can be detected with the pipeline of this detection kit. Accordingly, a fragment-capture method with a set of specific amplicons was recruited during library constructions. 50 ng DNA of each sample was used to generate sequencing libraries fully in line with the recommended protocols, before the sequencing process on HiseqX (Illumina, Inc., San Diego, CA, USA).

### 2.4. Data Processing and Statistical Analysis

Pretreatment and DNA bioinformatic analysis of the sequencing data of each sample was carried out on OncoAim™ cancer panel pipeline (OncoAim™ version 7.2) individually. Clean reads were assembled and aligned to reference data by the Burrow-Wheeler Aligner algorithm (https://github.com/lh3/bwa; version 0.7.12-r1039; December 2015). Unique reads derived from the GATK were used for variant calling. The minimum confidence threshold for variant and insertion/deletion (indel) calling was set to 0.05 (5%). The Integrative Genomics Viewer (version 2.3.94, Broad Institute) was used for visualization and confirmation of specific SNC/indel loci. Germline mutations were defined as follows: mutations found in both tumor and control DNA and/or mutations with a relatively high mutant allele frequency (MAF > 20%) and unconfirmed as somatic mutation in COSMIC (Catalogue Of Somatic Mutations In Cancer) database. Other mutations identified in our cohort were defined as somatic alterations. Additionally, statistical analysis and graphing were conducted using SPSS v19.0 and GraphPad prism 5. Significance was assumed for a *p* value of less than 0.05.

## 3. Results

### 3.1. Genetic Profiling of Melanoma Patients

We analyzed the gene variations of 39 melanomas from 27 patients. For all the NGS data in our study, the median uniformity was 98.66% (87.63%–99.85%) and most of the target coverages were over 1000x. Totally, 139 germline mutations in the 27 patients, 104 somatic variants, and 57 copy number variants in 39 tumors were revealed ([Supplementary-material supplementary-material-1], Supplementary data). No gene rearrangement was found. No difference in the germline, somatic alterations, or CNV was revealed between primary and metastasis melanomas. For each patient, the number of germline mutations ranged from 7 to 17 with a median of 11. The number of somatic mutations in most melanomas ranged from 0 to 9 except one sample with 14 variations. The specimen with most mutations harbored a gene alteration (T230M) in the SEMA domain of the MET gene, which mutated only in this melanoma. Besides, all the 14 variations observed in this sample were the unique mutations and not found in other samples.

### 3.2. Germline Mutation

FAN1 was the most common germline mutation gene in our cohort, of which 27 variations were found, followed by the genes EGFR, ERBB2, and MSH3, which were found mutated 20 times in melanomas of our study. For the mutations with a frequency above 50%, FAN1 G233E was observed in 25 samples, EGFR R521K in 20 samples, TP53 P72R in 19 samples, and ERBB2 P1140A in 15 samples. However, only one or no mutation was identified in the melanoma-susceptibility genes like CDKN2A and CDK4.

### 3.3. Somatic Mutation

The top gene variations were BRAF V600E (20.5%), NRAS Q61R (12.8%), RAD50 L580X (12.8%), TERT promoter (12.8%), and MSH6 T1085X (10.3%). More mutations were observed in tumors from female patients (*p* < 0.001).

**Figure 1:**
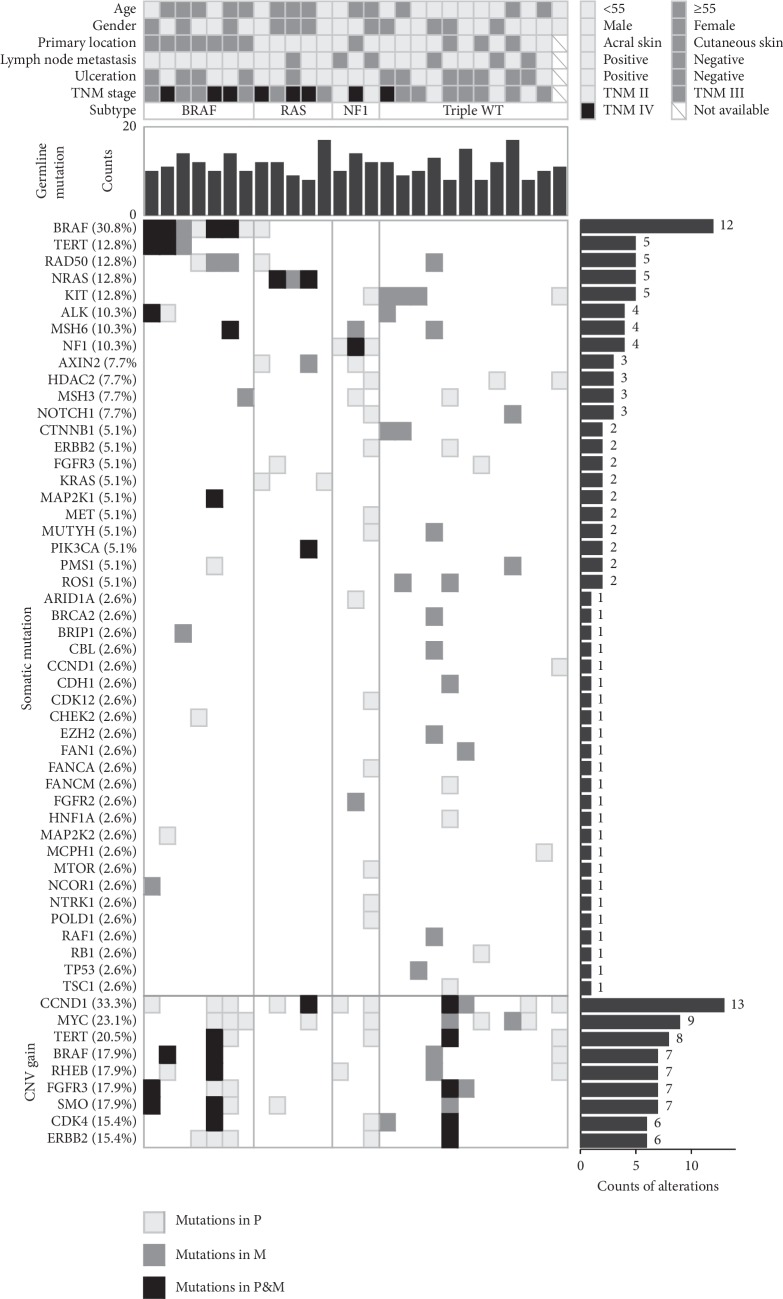
Landscape of genetic alterations in melanomas in China. The patient information, clinical characterizations, and molecular subtypes are presented for patients (top). (In one case listed in BRAF subtype, primary melanoma has a BRAF V600E whereas no mutation of BRAF/RAS/NF1 was detected in the paired metastasis melanoma; one case listed in RAS subtype harbored co-mutation of KRAS G12R and BRAF L567V.) The total counts of germline mutation, the matrix of somatic mutation, and CNV gain are indicated (middle and bottom). Mutations in different types of melanoma specimens (primary or metastasis) are shown with different fillings. Frequencies (left) and numbers (right) of gene mutation in our cohort are listed.

All the melanomas were classed into 4 group (BRAF, RAS, NF1, and Triple-WT) based on their major cancer driver genes. The number of the mutated gene BRAF was found in 12 melanomas from 8 patients. One tumor was identified as a KRAS (G12R) co-mutation with BRAF (L567V). Considering that this hotspot of KRAS has more clinical significance than that of the rare mutation in BRAF based on the knowledge at present, this melanoma was classed into the RAS group (Figure 1). So, there were 11 BRAF melanomas (11/39, 28.2%, 6 primary lesions; 5 metastatic lesions) from 8 patients. RAS gene mutated in 7 melanomas (7/39, 17.9%, 4 primary lesions; 3 metastatic lesions) from 5 patients. NF1 variants were observed in 4 samples (4/39, 10.3%, 3 primary lesions; 1 metastatic lesion) from 3 patients. We defined the Triple-WT subtype in 17 samples (17/39, 43.6%, 9 primary lesions; 8 metastatic lesions) from 13 patients (stage II: 3, stage III: 8, stage IV: 1, unknown: 1), within which KIT (4/17, 23.5%) took the most frequency among all these mutated genes. However, no mutation was detected in the other reported putative driver genes (FDGFRA, KDR, GNAQ, and GNA11) of the Triple-WT subgroup.

### 3.4. Copy Number Variation

In our study, CCND1 (13/39, 33.3%), MYC (9/39, 23.1%), and TERT (8/39, 20.5%) were the most common genes with CNV amplifications. For the tumor-associated genes ERBB2 and CDK4, amplifications were, respectively, found in 6 (15.4%) tumor samples of our cohort.

More CNV amplifications were detected in the non-CSD melanomas compared with those in acral melanomas (Table 1, *p*=0.004). No statistical correlations with gender, TNM, and lymph node status were found in our study (Table 1).

### 3.5. Distribution of Melanomas with Different Cancer Driver Genes

**Figure 2:**
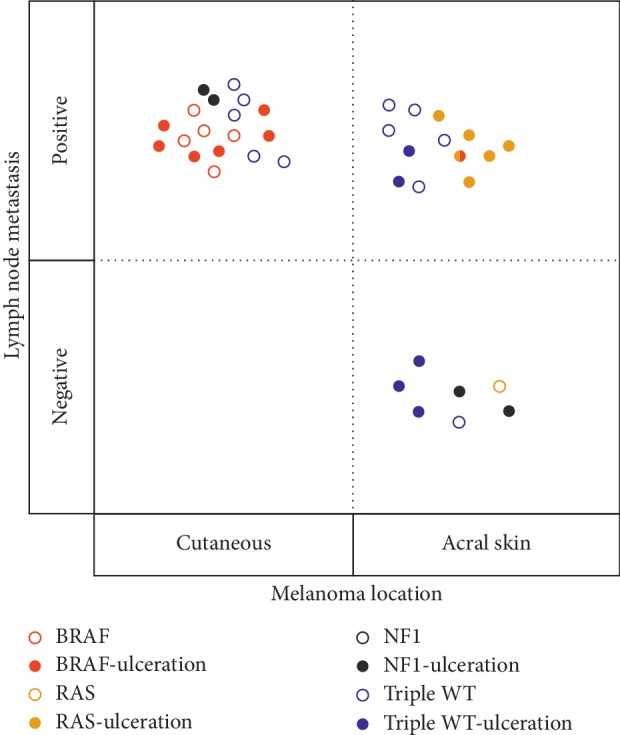
Clinical features in Chinese melanomas of 4 molecular subtypes. BRAF, RAS, NF1, and Triple-WT subtypes are indicated by different colors. One melanoma harboring co-mutation of BRAF and KRAS was indicated as half red and half yellow. Acral or cutaneous melanomas are indicated on the *X*-axis, melanomas with or without lymph node metastasis are indicated on the *Y*-axis, and melanomas with or without ulceration are indicated by the solid or hollow circles.

All the 11 samples in the BRAF group were observed in non-CSD cutaneous melanomas (11/18, 61%). One sample classified in the RAS group with BRAF mutation was observed in acral melanoma (1/20, 5%). Besides, all the patients with BRAF melanoma were found with lymph node metastasis (red dots in Figure 2).

All the 7 samples with RAS mutation were observed in acral melanoma (7/20, 35%). Patients with lymph node metastasis and/or ulceration had 6 RAS mutations, which was more than those without lymph node metastasis or ulceration (only one RAS mutation) (yellow dots in Figure 2).

All the NF1 mutations were observed in patients with ulceration (*p*=0.008, black dots in Figure 2). No interaction was revealed between Triple-WT mutations with tumor location, lymph node metastasis, and ulceration (blue dots in Figure 2 except one without pathological information).

## 4. Discussion

In the current study, we performed NGS sequencing with a multiple-gene panel to investigate the comprehensive molecular characterization of 39 melanoma tumors from 27 patients and evaluate the clinical correlations of gene status. Alterations in the detected genes showed no difference in the primary and metastatic lesions. Those mutations may be defined as a driver mutation in cancer tissues, which tend to be stable in metastases during the malignant process to maintain malignant phenotype as reported in literature [20, 21].

We analyzed the germline alterations in the DNA from melanoma patients to investigate if it contained known cancer-susceptibility gene mutations. However, few mutations were identified in the reported melanoma-susceptibility genes CDKN2A and CDK4. Germline mutations in the gene CDKN2A have been found in about 40% melanoma families and CDK4 alterations in 2-3% families [10]. It was reported [22] that acral melanoma is not associated with known melanoma-susceptibility genes like cutaneous melanoma. In our cohort, more than half the individuals were acral melanoma so that genetic variation patterns and family histories were quite different from Western countries in which acral melanoma was the rare subtype [4].

One primary melanoma was revealed with relative more somatic mutations than others. Much higher mutation burdens were discovered in many cancer types, such as melanoma [16, 23], colorectal cancer [24], lung cancer [25], and endometrial cancer [26]. A pathogenic gene alteration T230M was identified in the SEMA domain of the MET gene, which was reported in a sample of head and neck squamous cell carcinoma [27]. Previous reports suggest that mutations in the SEMA domain could activate the MET signal [27], which will promote tumor progress and more gene alterations.

In our study, CCND1, CDK4, and TERT showed a relatively high CNV gain frequencies, which was in agreement with the other cohort in published papers [6, 12–14]. Besides, the MYC gene was observed with CNV gains in more than 1/5 melanoma patients, which was not be reported before, implying a unique CNV status in Chinese melanomas. Previous studies indicated that cutaneous melanomas harbored the highest mutation burden due to DNA damage from UV radiation, and acral melanomas have a lower mutational load in Western countries [28]. However, similar somatic mutation frequencies were found in the acral and cutaneous melanoma cohort, which could explain that people of color consisting of Chinese mostly are less likely to be influenced by sunlight exposure [29] compared to the Caucasians in the West. Meanwhile, CNV amplifications of acral melanoma were significantly fewer than those of cutaneous melanoma, which seemed to suggest that the sunlight exposure affects the CNV rather than the mutations of Chinese melanomas. In our study, the incidence rate of somatic mutation of females was higher than that of males. In contrast, it has been reported that the incidence rate of mutation of females was lower than that of males for Caucasians in Western countries [30]. Ambient UV index appears to be associated with melanoma incidence in males but females in the US white population, due to the use of cosmetics and/or sunscreen in women against UV radiation [31]. Gender differences in somatic mutation showed different results in Chinese melanomas compared to Caucasians, which may be associated with the difference in the subtype of melanoma and lack of awareness of the skincare of Chinese women [32].

We divided our cohort into 4 subgroups based on the TCGA classification of melanoma [16]. As a previous study reported [33], the Triple-WT subgroup has the highest mutation rate in comparison to the BRAF^mut^, RAS^mut^, and NF1^mut^ subtypes. Within the Triple-WT group, KIT took the most frequent mutations and assumed to be an important oncogene like other studies [33, 34]. No other mutation was detected in the reported putative driver genes (KDR, GNAQ, and GNA11) in Western countries [18]. The NF1 subgroup has the lowest rate (10.3%) in our series, which were in accordance with those (9.1–14%) in the reported studies [33, 35]. The somatic mutations of the NF1 gene in the patients with ulceration were significantly higher than those in the patients without ulceration. Ulceration has been described to be an independent factor for poor prognosis in melanoma patients [36]. All of the melanomas in BRAF subtypes were observed in non-CSD cutaneous melanomas, which confirmed the conclusion of previous studies that highly prevalent of BRAF changes melanomas on skin intermittently exposure to ultraviolet (UV) irradiation, while BRAF mutations are rare on other skins including acral skin [6, 34, 37]. The frequency of BRAF mutations (61.1%) in non-CSD cutaneous melanomas was similar to those described in other publications as 55.2–66.7% in non-CSD [6, 33, 35, 37]. However, only one acral melanoma (5%) was observed in a non-V600E BRAF mutation, which was less than that (8.9–30%) in other cohorts [6, 33, 35, 37]. The less frequency of BRAF mutation may be associated with the arising sites of the acral melanomas. All the acral melanomas in our study were situated on palms and soles, on which lower frequency of BRAF mutations was demonstrated than that on dorsal acral sites [6, 35]. It is worth noting that the significance of BRAF mutation in melanomas is still a controversial issue. On the one hand, BRAF mutations were identified in about 80% benign naevi of various histological types [38], implying a critical role in the initiation of melanoma. On the other hand, BRAF mutations were found more common in melanomas with advanced stages like vertical growth phase, lymph node metastasis, or ulceration [7, 39, 40], suggesting that BRAF mutations correlated more with melanoma progression. All BRAF variants in our study were identified in patients with lymph node metastasis, which support the later statement that BRAF is important in progression rather than initiation of the melanoma. Unlike BRAF, all RAS mutations have been identified in acral melanomas. A relative low frequency of RAS mutations in non-CSD melanoma was reported in other Asian studies [33, 40] as well (2.0% in mainland China, and 5% in Taiwan island). Meanwhile, a higher frequency (22%) of RAS mutations in non-CSD melanoma was reported in a study based on patients worldwide [6]. These differences of genetic variation patterns may be associated with the differences in subtypes and ethnicities of melanoma. In the acral melanomas, RAS mutated more frequently in our cohort (35%) than 8.8–28% reported previously [6, 33, 35, 40]. As mentioned above, the palms and soles sites of the melanoma may contribute to the higher frequency, as higher frequencies of NRAS mutations were found in melanomas on the palms and soles rather than on the dorsal acral sites [6, 35]. Similar to NF1 and/or BRAF subtypes, patients with lymph node metastasis and/or ulceration had more RAS mutations than those without lymph node metastasis or ulceration. Based on the correlations between the driver gene (NF1, BRAF, and RAS) alterations and clinical characterizations (ulceration, lymph node metastasis), patients harboring more mutations were prone to poor prognosis.

## 5. Conclusion

In summary, mutational profiles of malignant melanomas in China are significantly different from Western countries. Lymph node metastasis and presence of ulceration were significantly associated with genetic alteration burdens. Our future studies will focus on clarifying the pathogenesis of melanoma and understanding the correlations of gene status with clinicopathologic characteristics in the Chinese population.

## Figures and Tables

**Table 1:** Clinical characterizations and gene alterations of the samples involved.

	No. of samples	CNV gains	Somatic mutations	No. of patients	Germline mutations
No. (percent)	39	32 (82.1%)	35 (89.7%)	27	27 (100%)

Total alterations		57	104		139

Age					
<55	20	80	55	13	152
≥55	19	107	49	14	155
*p* value		0.003	0.020		0.356

Gender					
Male	22	89	44	16	184
Female	17	98	60	11	123
*p* value		0.215	<0.001		0.677

Location					
Acral skin	20	73	49	15	169
Cutaneous skin	18	106	52	11	127
*p* value		0.004	0.482		0.610

TNM stage					
II	6	35	25	5	55
III	19	73	39	13	151
IV	13	71	37	8	90
*p* value		0.454^*∗*^	0.416^*∗*^		0.029^*∗*^

Lymph node metastasis					
Positive	29	131	70	19	218
Negative	7	35	26	6	64
*p* value		0.521	0.396		<0.001

Ulceration					
Positive	19	99	60	13	151
Negative	17	67	36	12	131
*p* value		0.315	0.656		0.051

^*∗*^These *p* values were based on the comparison in the overall.

## Data Availability

The data used to support the findings of this study are included within the article.

## Supplementary Materials

Supplementary Materials Supplementary table: the list of 223 genes in the OncoAim panel. Supplementary data: germline, somatic variations, and CNV gains identified by the NGS test in primary and metastasis melanomas from the Chinese population.

## References

[1] Siegel R. L., Miller K. D., Jemal A. (2017). Cancer statistics, 2017. *CA: A Cancer Journal for Clinicians*.

[2] Leichsenring J., Stögbauer F., Volckmar A.-L. (2018). Genetic profiling of melanoma in routine diagnostics: assay performance and molecular characteristics in a consecutive series of 274 cases. *Pathology*.

[3] Chi Z., Li S., Sheng X. (2011). Clinical presentation, histology, and prognoses of malignant melanoma in ethnic Chinese: a study of 522 consecutive cases. *BMC Cancer*.

[4] Bradford P. T., Goldstein A. M., McMaster M. L., Tucker M. A. (2009). Acral lentiginous melanoma: incidence and survival patterns in the United States, 1986–2005. *Archives of Dermatology*.

[5] Birkeland E., Zhang S., Poduval D. (2018). Patterns of genomic evolution in advanced melanoma. *Nature Communications*.

[6] Curtin J. A., Fridlyand J., Kageshita T. (2005). Distinct sets of genetic alterations in melanoma. *New England Journal of Medicine*.

[7] Bai X., Kong Y., Chi Z. (2017). MAPK pathway and TERT promoter gene mutation pattern and its prognostic value in melanoma patients: a retrospective study of 2,793 cases. *Clinical Cancer Research*.

[8] Read J., Wadt K. A. W., Hayward N. K. (2016). Melanoma genetics. *Journal of Medical Genetics*.

[9] Goldstein A. M., Chan M., Harland M. (2006). Features associated with germline CDKN2A mutations: a GenoMEL study of melanoma-prone families from three continents. *Journal of Medical Genetics*.

[10] Goldstein A. M., Chan M., Harland M. (2006). High-risk melanoma susceptibility genes and pancreatic cancer, neural system tumors, and uveal melanoma across GenoMEL. *Cancer Research*.

[11] Turajlic S., Furney S. J., Lambros M. B. (2012). Whole genome sequencing of matched primary and metastatic acral melanomas. *Genome Research*.

[12] Lin W. M., Baker A. C., Beroukhim R. (2008). Modeling genomic diversity and tumor dependency in malignant melanoma. *Cancer Research*.

[13] Hodis E., Watson I. R., Kryukov G. V. (2012). A landscape of driver mutations in melanoma. *Cell*.

[14] Kong Y., Sheng X., Wu X. (2017). Frequent genetic aberrations in the CDK4 pathway in acral melanoma indicate the potential for CDK4/6 inhibitors in targeted therapy. *Clinical Cancer Research*.

[15] Lyu J., Song Z., Chen J. (2018). Whole-exome sequencing of oral mucosal melanoma reveals mutational profile and therapeutic targets. *The Journal of Pathology*.

[16] Akbani R., Akdemir K. C., Aksoy B. A. (2015). Genomic classification of cutaneous melanoma. *Cell*.

[17] Cohen C., Zavala-Pompa A., Sequeira J. H. (2002). Mitogen-actived protein kinase activation is an early event in melanoma progression. *Clinical cancer research*.

[18] Hayward N. K., Wilmott J. S., Waddell N. (2017). Whole-genome landscapes of major melanoma subtypes. *Nature*.

[19] Palmieri G., Colombino M., Casula M., Manca A., Mandalà M., Cossu A. (2018). Molecular pathways in melanomagenesis: what we learned from next-generation sequencing approaches. *Current Oncology Reports*.

[20] Gibson W. J., Hoivik E. A., Halle M. K. (2016). The genomic landscape and evolution of endometrial carcinoma progression and abdominopelvic metastasis. *Nature Genetics*.

[21] Liao L., Ji X., Ge M. (2018). Characterization of genetic alterations in brain metastases from non-small cell lung cancer. *FEBS Open Bio*.

[22] Phan A., Touzet S., Dalle S., Ronger-Savlé S., Balme B., Thomas L. (2007). Acral lentiginous melanoma: histopathological prognostic features of 121 cases. *British Journal of Dermatology*.

[23] Ding L., Kim M., Kanchi K. L. (2014). Clonal architectures and driver mutations in metastatic melanomas. *PLoS One*.

[24] Gong J., Cho M., Sy M., Salgia R., Fakih M. (2017). Molecular profiling of metastatic colorectal tumors using next-generation sequencing: a single-institution experience. *Oncotarget*.

[25] Govindan R., Ding L., Griffith M. (2012). Genomic landscape of non-small cell lung cancer in smokers and never-smokers. *Cell*.

[26] Briggs S., Tomlinson I. (2013). Germline and somatic polymerase *ϵ* and *δ* mutations define a new class of hypermutated colorectal and endometrial cancers. *The Journal of Pathology*.

[27] Seiwert T. Y., Jagadeeswaran R., Faoro L. (2009). The MET receptor tyrosine kinase is a potential novel therapeutic target for head and neck squamous cell carcinoma. *Cancer Research*.

[28] Goodman A. M., Kato S., Bazhenova L. (2017). Tumor mutational burden as an independent predictor of response to immunotherapy in diverse cancers. *Molecular Cancer Therapeutics*.

[29] Gupta A. K., Bharadwaj M., Mehrotra R. (2016). Skin cancer concerns in people of color: risk factors and prevention. *Asian Pacific Journal of Cancer Prevention*.

[30] Gupta S., Artomov M., Goggins W., Daly M., Tsao H. (2015). Gender disparity and mutation burden in metastatic melanoma. *Journal of the National Cancer Institute*.

[31] Liu-Smith F., Farhat A. M., Arce A. (2017). Sex differences in the association of cutaneous melanoma incidence rates and geographic ultraviolet light exposure. *Journal of the American Academy of Dermatology*.

[32] Cheng S., Lian S., Hao Y. (2010). Sun-exposure knowledge and protection behavior in a North Chinese population: a questionnaire-based study. *Photodermatology, Photoimmunology & Photomedicine*.

[33] Sheen Y. S., Tan K. T., Tse K. P. (2019). Genetic alterations in primary melanoma in Taiwan. *British Journal of Dermatology*.

[34] Curtin J. A., Busam K., Pinkel D., Bastian B. C. (2006). Somatic activation of KIT in distinct subtypes of melanoma. *Journal of Clinical Oncology*.

[35] Zaremba A., Murali R., Jansen P. (2019). Clinical and genetic analysis of melanomas arising in acral sites. *European Journal of Cancer*.

[36] Zhang M., Zhang N. (2017). Clinical and prognostic factors in 98 patients with malignant melanoma in China. *Journal of International Medical Research*.

[37] Ashida A., Uhara H., Kiniwa Y. (2012). Assessment of BRAF and KIT mutations in Japanese melanoma patients. *Journal of Dermatological Science*.

[38] Pollock P. M., Harper U. L., Hansen K. S. (2003). High frequency of BRAF mutations in nevi. *Nature Genetics*.

[39] Dong J., Phelps R. G., Qiao R. (2003). BRAF oncogenic mutations correlate with progression rather than initiation of human melanoma. *Cancer Research*.

[40] Si L., Kong Y., Xu X. (2012). Prevalence of BRAF V600E mutation in Chinese melanoma patients: large scale analysis of BRAF and NRAS mutations in a 432-case cohort. *European Journal of Cancer*.

