# Supplementary material for: Characterizations of Gene Alterations in Melanoma Patients from Chinese Population
Source: Biomed Res Int. 2020 Jan 30;2020:6096814. doi: 10.1155/2020/6096814 (PMC7011309; doi:10.1155/2020/6096814)
Supplement: Supplementary Materials — Supplementary table: the list of 223 genes in the OncoAim panel. Supplementary data: germline, somatic variations, and CNV gains identified by the NGS test in primary and metastasis melanomas from the Chinese population. [file 6096814.f1.zip › 6096814.f1/supp table 1 6096814.v1.pdf]

Supplementary Table: The list of 223 genes in the OncoAim panel.

1） SNV & InDel									
ABCB1	ABCC4	ABCG2	ABL1	ABL2	AFF3	AKT1	ALB	AR	ARAF
ARID1A	ARID2	ATR	AXIN2	BARD1	BCL2L11	BLM	BMP3	BMPR1A	BRIP1
BTK	C8orf34	CARD11	CBL	CBR3	CCND1	CCND2	CCNE1	CD274	CDA
CDH1	CDK12	CDK4	CDK6	CDKN2A	CHEK1	CHEK2	CLIP1	CREBBP	CRLF2
CSF1R	CSF3R	CTNNB1	CUL3	CYP19A1	CYP1B1	CYP2B6	CYP3A5	DDR2	DHFR
DNM2	DNMT3A	DPYD	DYNC2H1	EMSY	EPHX1	ERBB3	ERBB4	ERCC1	ERCC2
ERCC3	ESR1	ETS2	EZH2	FAM175A	FAN1	FANCA	FANCC	FANCD2	FANCF
FANCL	FANCM	FBXW7	FCGR2A	FCGR3A	FGFR4	FLT3	FOLR3	FOXA1	FOXL2
GALNT12	GATA3	GGH	GNA11	GNAQ	GNAS	GREM1	GSTP1	HDAC2	HGF
HNF1A	HOXB13	HRAS	IDH1	IDH2	IGFBP7	JAK1	JAK2	JAK3	KDR
KEAP1	KIT	KRAS	MAP2K1	MAP2K2	MAP2K4	MAP3K1	MAPK1	MAPK3	MCPH1
MECOM	MED12	MEX3A	MLH3	MLL3	MPL	MRE11	MSH3	MSH6	MTHFR
MTOR	MTR	MUTYH	MYC	MYCN	MYD88	NBN	NCOR1	NF1	NF2
NFE2L2	NOTCH1	NPM1	NQO1	NRAS	NRG1	NT5C2	NTHL1	NTRK3	PALB2
PCBP1	PDE4DIP	PDCD1	PDCD1LG2	PDGFRA	PMS1	PMS2	POLD1	POLE	PPP2R1A
PPP2R2A	PTCH1	PTPN11	Q7Z2S2	RAC1	RAD50	RAD51	RAD51B	RAD51C	RAD51D
RAD54L	RAF1	RB1	RHEB	RHOA	RIT1	RNF43	RPL22	RPS20	SEMA3C
SETBP1	SF3B1	SFRP1	SFRP2	SLC19A1	SLC22A16	SLC28A3	SMARCB1	SMO	SOCS1
SOD2	STAG2	SULT1A1	TCF3	TERT	TFPI2	TPMT	TSC1	TSC2	TYMS
U2AF1	UGT1A1	UMPS	VHL	XPC	XRCC1	XRCC2			
1) SNV, InDel and CNV									
APC	ATM	BRCA1	BRCA2	EGFR	EPCAM	ERBB2	FGFR1	MET	MLH1
MSH2	PIK3CA	PTEN	SMAD4	STK11	TP53				
2) SNV, InDel, CNV and Fusion									
ALK	FGFR2								
3) SNV, InDel and Fusion									
BRAF	FGFR3	NTRK1	RET	ROS1					
4) Fusion									
PPARG	THADA	TMPRSS2							
